# Social, political and legal determinants of kidney health: Perspectives from lower- and middle-income countries with a focus on India

**DOI:** 10.3389/fneph.2022.1024667

**Published:** 2022-10-24

**Authors:** Urmila Anandh, Priti Meena, Sabine Karam, Valerie Luyckx

**Affiliations:** ^1^ Department of Nephrology, Amrita Hospitals, Faridabad, Delhi NCR, India; ^2^ Department of Nephrology, All India Institute of Medical Sciences, Bhubaneshwar, India; ^3^ Department of Medicine, University of Minnesota, Minneapolis, MN, United States; ^4^ University Children’s Hospital, Zurich, Switzerland

**Keywords:** poverty, climate change, gender inequity, dialysis, kidney health

## Abstract

The social determinants of health (SDoH) are the non-medical factors that influence kidney health outcomes directly or indirectly in a substantial manner and include conditions in which people are born, grow, work, live, and age. Many such challenges in lower- and middle- income countries have an unfavourable impact on kidney health. These conditions potentially influence economic policies and systems, development agendas, social norms, social policies, and political systems. In addition, many political and legal factors also determine and modify the ultimate outcome in patients with kidney disease. Legal factors that ensure universal health care, promote gender and racial equality, prevent malpractices and regulate strict laws in the field of kidney transplantation are the paramount determinants for the provision of necessary kidney care. Converging lines of evidence have supported the impact of social variables such as socioeconomic resources, social inclusion, housing conditions, educational attainment, and financial status on kidney health, particularly affect vulnerable and disadvantaged groups and result in challenges in kidney care delivery. Furthermore, the climate is an important SDoH that plays a crucial role in the occurrence, prevalence, and progression of kidney diseases as highlighted by the presence of higher prevalence of chronic kidney disease in hot tropical countries. The rising incidence of water and vector-borne diseases causing acute kidney injury is another consequence of disruptive environmental and climate change which is detrimental to kidney health. Political risk factors such as conflict also have a devastating influence on kidney health. The relationship between SDoH and kidney health outcomes requires more clarity. Gaps in the current knowledge need to be identified to inform the development of appropriate interventions to address upstream socio-economic risk factors for kidney disease.

## Introduction

During the past two decades, increasing evidence has highlighted the strong relationship between social determinants of health (SDoH) and health outcomes. As per the World Health Organisation (WHO), SDoH are the “conditions in which people are born, grow, live, work, and age.” (1) Socioeconomic conditions, psychosocial factors, environmental factors, and cultural-political drivers are the main domains of SDoH. Variables such as poverty, education attainment, unemployment, housing and working conditions, racial discrimination, and cultural beliefs are the key mediators of the overall health status of any country. Social and environmental factors are highly consequential and can result in remarkable variation in health status (2). The SDoH influence implementation of many social policies associated with medical care that have the potential to alter health-related outcomes (3). Lower educational attainment can make it difficult for the patients to navigate the complex healthcare system. Utilization and access to primary health care are impeded by a lack of health insurance causing roadblocks in detecting and treating risk factors for kidney disease, such as hypertension and diabetes mellitus. SDoH also foster an environment that perpetuates kidney health inequities. Disparities related to SDoH have a key role in the progression of chronic kidney diseases (CKD), its management, and as a risk factor (4). Legal factors (the laws of the local government addressing socio-economic, gender, and ethnic inequities) and the political climate of the country (policies of the government, its interaction with various international agencies, presence of conflict, etc.) affect the social determinants of kidney health. This review aims to address the primary domains of SDoH with their implications on kidney health and provides a focus on how the enhanced understanding of the SDoH can be utilized to improve treatment plans and kidney outcomes.

**Figure 1:**
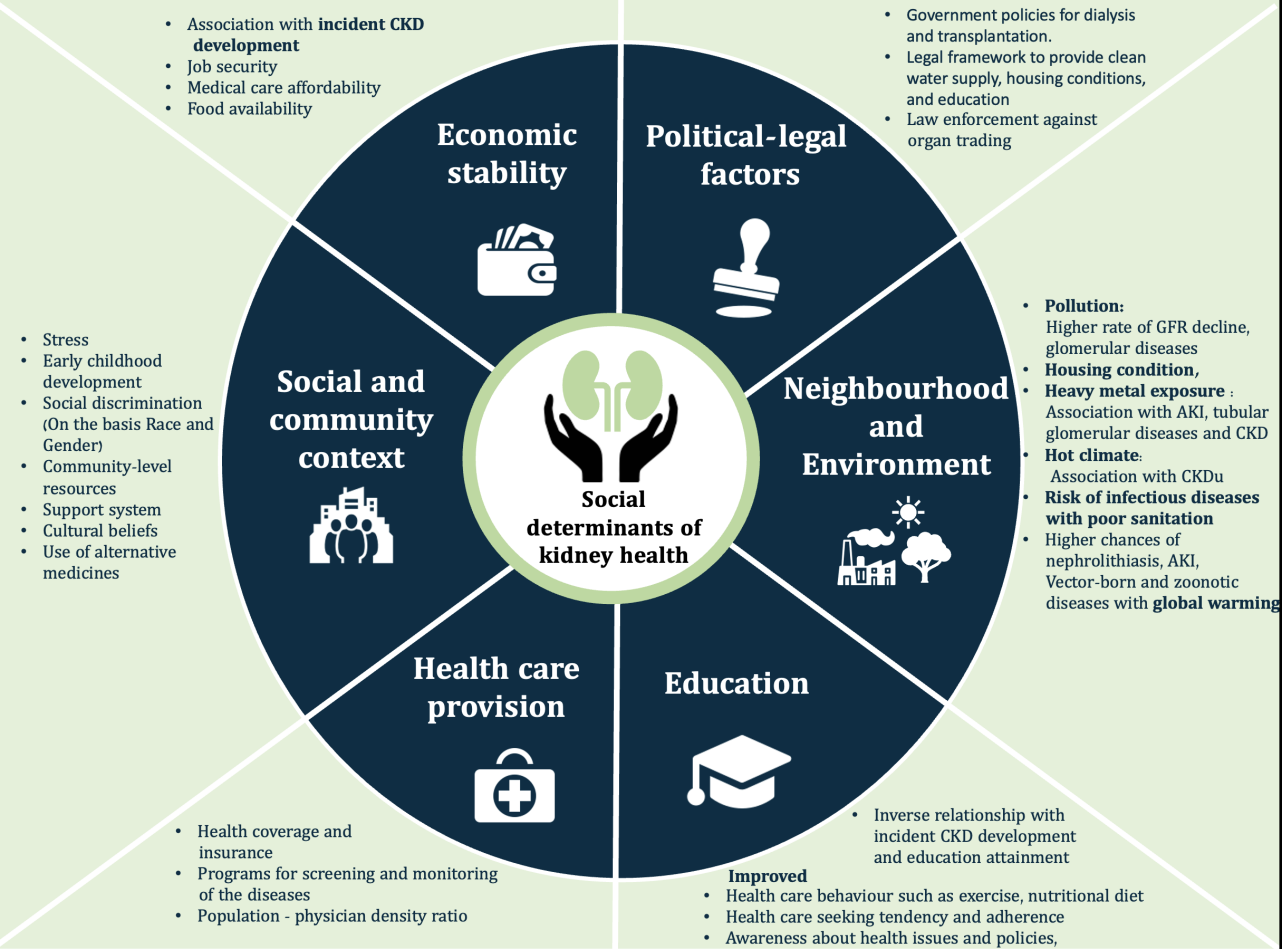
The social determinants of health. AKI, Acute kidney injury; CKD, Chronic kidney disease; CKDu, Chronic kidney disease of undetermined origin; GFR, Glomerular filtration rate.

Key Social determinants of health are summarized in **Figure 1**.

## Social determinants of health

### A: Socio-economic status and kidney disease

Economic status is one of the most significant domains of the SDoH. Current data shows that approximately 22% of the world’s population residing in 107 developing countries is affected by multidimensional poverty (5). Around 85% of the population affected by poverty resides in sub-Saharan Africa and South Asia (5). Education, unemployment, social security, food insecurity, inability to pay for medical care, income, and living conditions are the important domains of poverty (6). There is a strong association between low economic status and prevalence and outcomes of kidney diseases (7), as poverty is the main hindrance to accessing health care. Reduced access to healthcare facilities, non-affordability, limited follow-up care, and delayed treatment have considerable ramifications for adults and children living under poor socioeconomic conditions (8, 9). Even in high-income settings such as Canada, children aged four to 11 years from lowest-income families had a 2.5 times higher risk of impaired functional health (e.g., vision, hearing, speech or mobility) as compared to highest-income families (10). Poverty is also likely contributing to the swift rise in chronic diseases such as diabetes mellitus and cardiovascular diseases being observed in Low and Low Middle-Income Countries (LMIC) and Middle-Income Countries (MIC) (11). In India, the uneven delivery of kidney healthcare, especially in children, where the rates of morbidity and mortality are dependent on the socio-economic status of the parents raises many moral and ethical issues (12). Similarly, de facto rationing of dialysis in Africa is related to poverty. The moral distress of all stakeholders - patients, families, and clinicians -resulting from the inability to access or deliver care must be addressed and acknowledged (13). It is high time for a global call to action against poverty. Legal and political frameworks must be developed to expand programs within existing social assistance systems to tackle poverty and mitigate poverty’s effect on kidney health.

Another SDoH is malnutrition. Socioeconomic status and malnutrition are intertwined. The prevalence of malnutrition is higher in low- and middle-income countries (14).

Malnutrition has both direct and indirect effects on kidney health. Maternal nutrition directly influences the kidney development of the offspring. Perturbations to maternal nutrition during pregnancy are shown to be linked with a higher likelihood of metabolic, cardiovascular, and kidney disease in the offspring (15–17). A high prevalence of protein-energy malnutrition (PEM) is seen in patients with advanced CKD and those on dialysis. PEM is also accompanied by a high inflammatory process. The “malnutrition-inflammation complex syndrome” (MICS) has a strong association with atherosclerotic cardiovascular disease. MICS is mostly attributed to factors such as reduced dietary intake, nutrient loss in dialysis, metabolic acidosis, oxidative stress and protein breakdown. Malnutrition in CKD patients is prevalent in both developed and developing countries and is a substantial cause of mortality and morbidity (18). It is, therefore, necessary to advocate for improving food security for the vulnerable and underprivileged population. In one such instance, “The Uganda Food and Nutrition Policy in Uganda” is promoting nutritional status through multi-sectoral and coordinated interventions to provide food security and better nutrition for the population (19).

### B: Education and kidney disease

Several studies have shown an association between incident CKD development and educational attainment (20). In the Indian CKD cohort, a significant percentage of patients were illiterate (26.9%). This subgroup of patients, when asked about simple aspects of kidney disease (symptoms, availability of treatment etc) had lower levels of awareness about CKD (21).

In another analysis, an allele score–based Mendelian randomization indicated that higher education attainment was associated with a decreased CKD risk (22). In the German CKD (GCKD) cohort higher all-cause mortality, and poor kidney and cardiovascular outcomes were reported in people with low educational attainment (23). The potential effect of education status on kidney disease warrants targeted public interventions by government and policymakers to mitigate the growing burden of the CKD population. The “National Education Policy” in India focused on providing universal access to school education, is certainly a step forward on this issue (24).

### C: Housing conditions and kidney disease

Homelessness and unstable housing are well-established determinants that result in adverse health consequences (25, 26). Housing insecurity is described as unaffordable housing payments and unsafe or overcrowded living conditions that impede general self-care and independence (27). Apart from the increased risk of infectious diseases in overcrowded living conditions, poor housing conditions are associated with non-communicable chronic diseases such as diabetes, and hypertension (28).

Individuals residing in LIC and LMIC undergoing rapid urbanisation have faster kidney function decline (29). In the US, housing insecurity was also linked to subsequent albuminuria at a median follow-up of 3.5 years (30). A study from San Francisco revealed that homeless adults were at 1.28 times greater risk of death or kidney failure than individuals with stable housing (31). Neglected medical care along with a stressful lifestyle results in recurrent hospitalization, increased disease progression, and higher mortality. People with unstable housing often miss their dialysis sessions (32).

The stress of patients with kidney disease is augmented by other factors related to improper housing conditions such as poor water quality, higher occurrence of infectious diseases, mental health problems, and substance abuse (26).

Addressing housing is an important SDoH and managing it with advocacy and program development is imperative. Housing and human settlements development policies in different countries have already made tremendous progress in facilitating sustainable housing development, and extending such policies to kidney patients might help to overcome unstable housing issues in such patients (33).

### D: Gender disparities

Gender discrimination in society drives inequalities in healthcare. Gender disparity has resulted in unequal health opportunities, differential health-seeking behaviours, vulnerabilities to diseases, and biases in medical research (34). There is an ongoing failure to maintain gender equality in the field of research. For instance, in a study from a metropolitan city in south India, of the 2158 participants in an educational and screening program for CKD amongst youth, only 32% were females (35). Gender-specific differences are observed in the aetiology, progression, and epidemiology of CKD. A higher prevalence of CKD and a slower rate of progression occurs in females as compared to males (36). However, females face more challenges like late nephrology referrals and delayed initiation of dialysis (37). And, even when initiated on dialysis women are less likely to receive optimal dialysis (38). It is not yet clear in lower-income settings, if the differences in RRT initiation between men and women are related purely to differences in the rate of CKD progression or are also impacted by nonbiological factors, such as unequal access to care or personal preference (39). What is clear, however, is that the quality of dialysis care in women, however, appears to be lower. For example, the prevalence of anaemia and malnutrition is higher in female CKD patients (40). Women also have lower odds of having arteriovenous (AV) access as compared to males for hemodialysis initiation (41). The gender-specific disparities are partly explained by the fact that women are less frequently employed, have other social responsibilities and often give low priority to their health (42). Population studies had shown that a substantial proportion (up to 27%) of women of reproductive age especially from areas like Africa may have the presence of pre-existing risk factors for renal disease such as hypertension that may be revealed during the physiological stress period of pregnancy (43, 44).

Studies have projected that elderly women opt more for conservative treatment rather than dialysis (45). Women are also disadvantaged as far as transplantation is concerned - the majority of living kidney donors are females, yet they are less likely to receive a kidney transplant (46). In various kidney transplant waiting list databases, there are fewer women than men (47). In a study from India analysing more than 5000 transplants women fare very poorly. They form the major donor pool and hardly ever receive an organ. In this study, only 32% of deceased donor kidney transplant recipients were females (48). This disparity is universal (49) but much more pronounced in African and Asian countries with socio-cultural practices, higher economic dependency and higher illiteracy rate in women being the most plausible explanations (50).

Autoimmune diseases preferentially affect women and are major causes of morbidity in females (51). Adverse effects on reproductive health and pregnancy are another pressing issue for female CKD patients. There is an increased risk of pre-eclampsia, premature delivery, and fetal complications (52).

Government policies like Janani Shishu Suraksha Karyakaram in India are a step forward in overcoming difficulties faced by pregnant women by providing completely free services to pregnant women (53). Supervised deliveries and timely management of complications in pregnant women can halt and prevent further complications like pregnancy-related acute kidney injury (AKI). Women with or at risk of CKD can be identified during pregnancy and referred for follow-up. Offspring of these higher risk pregnancies should also be identified and received life-long follow up. Mitigation of gender disparity in nephrology does not only involve enhancing education or poverty alleviation, but it needs a deeper and vigorous analysis of traditional gender roles and the place of a woman in society. An equitable deceased donor allocation system that prioritizes female recipients, especially those who are difficult to match requires discussion, but may be an example of a policy that could address current disparities.

### E: Kidney health and discrimination based on race and ethnicity

Worldwide, discrimination because of race and ethnicity is inextricably related to adverse health outcomes. Racism confers inequalities in opportunities, income, access to health care, employment, education, health insurance, food insecurity, and community-level resources (54). For the majority of patients in the United States, kidney replacement therapy (KRT) is covered by Medicare, but this facility is not covered for undocumented immigrants (55). Accumulating evidence suggests that African Americans and Hispanics are at a 2.6 and 1.5-fold, respectively higher risk of developing kidney failure and have faster progression than white individuals (56). There is an inequitable higher prevalence of co-morbidities like diabetes and hypertension in these populations (57).

Besides genetic predisposition (58), adverse kidney outcomes in African Americans are further driven by late nephrology referrals. Most of them initiate hemodialysis with a catheter, rather than a surgically placed AV fistula (AVF). In a cohort of 396,075 patients, white patients were more likely to be initiated on hemodialysis with an AVF as compared to black patients or Hispanic patients (18.3% vs 15.5% and 14.6%, respectively; P < .001) (59) They are also less likely to receive kidney transplantation compared to Caucasians (60).

This disadvantage is not restricted to those of African origin, even indigenous ethnic minorities cannot access suitable and optimal healthcare. This is reflected in the higher incidence of kidney disease and poorer access to healthcare in indigenous populations (61). The life expectancy is lower in indigenous Australians and even now there are limitations in accessing healthcare for vulnerable groups such as the Aboriginal and Torres Strait Islander (indigenous) population (62). Subtle racial discrimination is also present in India where a complex caste substructure exists in the society. People are divided into castes which historically indicated their vocation. With the development of “superior” and inferior” vocations, Indians of the lower castes still have limited access to healthcare, especially in rural India (63). Also in India, there is a discrepancy in life expectancy at birth as there is a complex interplay between caste, religion and economic development of the region (64). Caste system is not uncommonly inextricably linked to socioeconomic status. Even the benefits of caste-based jobs, education opportunities, and health-related schemes are mostly availed by better-off people within the caste group. Despite improvements and multiple affirmative actions, the life expectancy of the lower castes is still lower. Studies are required to see whether this caste system has any impact on kidney health in Indians.

Another area where race impacts kidney care is the misclassification of the estimated glomerular filtration rate (eGFR) due to the race coefficient. The inclusion of race in the eGFR equation had indirectly deprived African Americans of timely nephrology care especially at advanced stages, compounding the disadvantage (65). Excluding race from eGFR calculations is necessary to address the impact of structural racism on patients with kidney diseases and improve clinical care and outcomes (66).

### F: Environment hazards (climate change) and kidney health

The footprints of unrestrained human activities such as deforestation, pollution, and rapid urbanization are progressively causing a rise in global temperature (67). Rising temperatures pose a huge threat to human health (68). As a consequence of climate change, the incidence of kidney disorders is also expected to rise globally. Hot weather causes an increase in core body temperatures resulting in dehydration, rhabdomyolysis, hyperosmolality, and inflammation-mediated recurrent kidney injury (69). A higher frequency of urinary tract infections and nephrolithiasis have also been reported with global warming (70). In recent years, the world is facing another pressing challenge chronic kidney diseases of unknown origin (CKDu) (71, 72). The cases of heat stress nephropathy have been reported worldwide (73–75). The renal histology usually shows tubulointerstitial nephritis (76).

**Figure 2:**
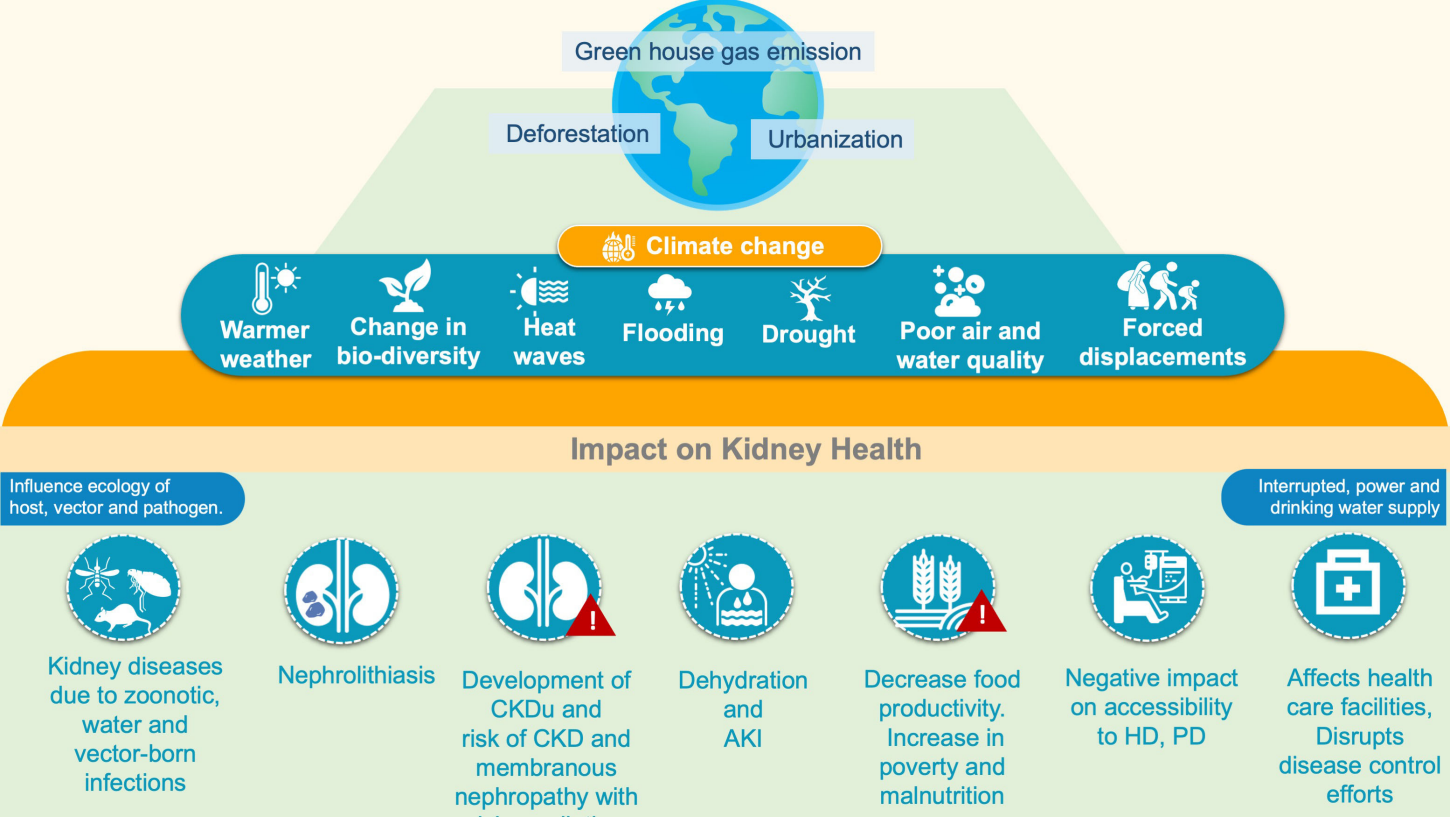
Climate and kidney Health. AKI, Acute kidney injury; CKD, Chronic kidney disease; CKDu, Chronic kidney disease of undetermined origin; GFR, Glomerular filtration rate; HD, Hemodialysis; PD, Peritoneal dialysis.

Climate change and environmental disruptions have also been increasingly connected to a higher frequency of infectious diseases (77). Increased extreme weather events (floods, drought etc) are associated with increased incidences of vector-borne and zoonotic diseases (leptospirosis, malaria, dengue, hantavirus nephropathy scrub typhus, diarrheal illness etc) which consequently have a detrimental impact on kidney health (78). These infections are the most common preventable causes of AKI typically in tropical and subtropical regions (79) (**Figure 2**)

Epidemiological evidence suggests the role of environmental toxins in kidney diseases. Heavy metals like lead, mercury, arsenic, and cadmium are associated with CKD, AKI, tubular disorders (Fanconi syndrome), and glomerular diseases (80). Kuźma et al. demonstrated an association between eGFR decline and CKD development with medium- and short-term exposure to higher air pollution levels (81). Continuous efforts are required to prevent further environmental degradation, including improving sanitation and reducing pollution worldwide (82).

## Political determinants of health

The most obvious example of politics impacting kidney health is the risk imposed by residence of patients in areas of conflict. This is becoming increasingly relevant as many parts of the world are enmeshed in civil wars, external invasions and humanitarian crises, often superimposed on pre-existing challenges in LIC and LMIC countries. Persistent long drawn conflicts not only affect the overall physical and mental health of its citizens, but also make delivery of care extremely challenging as the infrastructure becomes heavily affected. In Northwest Syria for example, as a result of the conflict, by 2020 more than half of health facilities had closed and many others were being attacked (83), while in in the Tigray region of Ethiopia, six months into the war that started in November 2020, only 27.5% of hospitals and 17.5% of health centres were still functional (84) Moreover, in that region, war and blockade have caused reduced access, funding and severe supply shortages to the only hemodialysis facility that serves a population of 9,000,000 people, condemning many patients with treatable illnesses to die (85). In addition, wars can lead to the exodus of skilled healthcare workers as they can be directly targeted by weapons (86). A notorious example is the Syrian conflict which has been estimated to have led to the exodus of more than 70% of the healthcare workforce (87). Furthermore, humanitarian crises and unstable political environments can also drive emigration as in Venezuela where 30000 physicians of which 150 are nephrologists have migrated to other countries over the past five years (88) and in Lebanon where the ongoing economic crisis coupled with the Beirut port explosion has already driven at least 1000 out of 15000 physicians out of the country (89). For those who remain, burnout can be a serious concern and constitute an additional barrier to optimal care delivery (87, 90). Moreover, the lack of adequate infrastructure and workforce can weaken the medical education system and impede both its graduate and post-graduate components, sabotaging not only the present but also the future of healthcare (87). In Iraq, a country that has long suffered of political instability, a recent cross-sectional survey identified that most medical students intended to leave the country after graduation (91). Finally, massive population displacement could also disrupt the healthcare care systems of the host countries, jeopardize the well-being of both the refugees and the local population and foster outbreaks of different types of diseases (92). A qualitative study conducted in Lebanon among nurses and nursing directors in regions in regions with a high concentration of Syrian refugees, identified as repercussions of their influx, fatigue, burnout, and depleted compassionate care at the individual level; rationing and stressed interpersonal relationships at the practice level and shortage in resources and poor performance at the healthcare system level (93). The war in Ukraine has led to the fastest number of migrating refugees since World war II (https://data.unhcr.org/en/situations/ukraine). Both their number and their rapid influx have made it challenging for neighboring countries to provide the adequate medical services not only for the migrants but for locals as well (94). Therefore, the World Health Organization (WHO) has recognized that maintainable health is largely determined by policies that guide actions beyond the health sector and has called all its members to integrate Health as part of policies directed at achieving sustainable development goals such as education, economy, transport, and housing (https://www.who.int/activities/promoting-health-in-all-policies-and-intersectoral-action-capacities).

## Legal determinants of health: Government policies and laws affecting kidney health in India

Most of the social factors impacting kidney health are modifiable and can be improved upon. Housing, gender disparity, education, and socio-economic inequities are in a continuous flux the world over. The regional differences in the magnitude of the impact of social determinants on kidney health is not only an issue to be addressed by the healthcare officials, but is also a responsibility of the state. Nations can do a lot in improving health of its citizen by promulgating laws protecting various aspects of the “right to health”. All nations have promulgated laws ensuring “Universal Health Care” (UHC). But many lower and middle- income countries lag behind in offering basic health care to its populace. In addition, there are layers of discrimination in the basic delivery of health care. Governments also fail to provide any legal protection to the most under served population (95). To achieve UHC, there are three core legal determinants-a) health laws should address all social determinants of health, b) health and ancillary systems should be well governed, and c) public health officials should abide by the law (96). A strong legal protection to the right to health also has a major impact on kidney health as a strong legal framework is responsible for just and equitable access to treatment of kidney diseases (97). In the Indian perspective, various policies and programs are in place (some with legal protection) which have made a difference (both directly and indirectly) in promotion of kidney health (**Table 1**). An instance wherein a strong legal system has impacted kidney health positively in India is the rigorous implementation of the medical termination of pregnancy (MTP) act. This has led to a drastic reduction in illegal abortions which in turn has had a salutary impact on the incidence of pregnancy related acute kidney injury (105). However, as a nation, India and other LMICs have a long way to go in mitigating various social, political and legal factors which directly and/or indirectly affect kidney health (106).

**Table 1:** Social determinants and Government Policies: The Indian Perspective.

Social determinants	Government policies	Comments
Universal Health Coverage and Chronic Kidney Disease	Financial Protection to patients with CKD (Government of Andhra Pradesh) (98) The National Dialysis Programme (2016) (99)	Financial support to CKD patients is miniscule as renal replacement therapy is prohibitively expensive. The Indian Government is committed to start an eight -station dialysis unit in each of the 688 districts offering free hemodialysis. The programme has been extended to include peritoneal dialysis.
Gender Equality	The government of India has many departments and policies for women 1.Ministry of Women and Children 2.National Policy for the Empowerment of Women 3.National Commission for Women.	These governmental bodies enact various laws including the medical termination of pregnancy (MTP) act (100) and the pre-conception and pre-natal diagnostic techniques (PCPNDT) act (101). Some of these initiatives have made an impact on kidney health (53). Despite policies, the gender gap in India is widening.
Environmental determinants (Sanitation,Water)	National Sanitation Policy (102) National Flourosis Mitigation Programs (103)	Various community participatory initiatives in urban sanitation are slowly showing theirs benefit. The national programme has reduced the incidence of fluorosis.Reduction in the fluoride content of water has led to reduction in CKD incidence in certain parts of the country.
Climate Change	The Government of India has a strong focus on climate change and runs many programs (104). These include- 1.National Solar Mission National Mission for Enhanced Energy Efficiency National Mission on Sustainable Habitat National Water Mission Green India Mission etc.	These programs are in their formative years and it is hoped that there will be a positive impact on the climate and consequently on kidney health.

**Table 2** summarizes impact of each SDoH on kidney health outcomes and adaptive measures.

**Table 2:** Impact of each SDoH on kidney health outcomes and adaptive measures.

SDoH	Impact	Adaptive measure
**Health Care system**	•Impact on hemodialysis and peritoneal dialysis services. •Quality of health care. •Health care accessibility	•Screening for diseases like diabetes mellitus, hypertension kidney dysfunction in the high-risk population. •Improve physician-patient density. •Provision of medical insurance for dialysis. •Improving access to the health care system
**Education**	•Poor education status has been linked with CKD development	•Development of government policies focusing on providing universal access to school education.
**Social and community context**	•Racial and gender discrimination •Cultural beliefs •Use of alternative medications	•Policy development for the provision of equity in the health care system (Without racial and gender bias) •Encouragement of community support and social integration. •Creating awareness and providing knowledge about treatment options.
**Poverty and unemployment**	•Food insecurities and housing insecurities are associated with incident CKD development. •Malnutrition causes substantial mortality and morbidity in CKD patients. •Low SES causes reduced access to healthcare facilities, non-affordability, limited follow-up care, and delayed treatment. •Maternal nutrition during pregnancy is associated with a higher likelihood of metabolic, cardiovascular, and kidney disease in the offspring	•Improving food housing and security, particularly for vulnerable (including patients with kidney diseases) and underprivileged populations. •Providing more job opportunities with health insurance (including dialysis services). •Development of policies to provide proper maternity care (Free antenatal and postnatal care provision, focus on maternal nutrition and co-morbidities)
**Environment exposures**	•Association of rising pollution with a higher rate of GFR decline and glomerular diseases •Housing condition: Heavy metal exposure have been reported to be associated with AKI, tubular •glomerular diseases and CKD •Hot climate is implicated in causative factors of CKDu •Higher risk of infectious diseases with poor sanitation and overcrowding. •Higher incidences of nephrolithiasis, AKI, Vector-born and zoonotic diseases with global warming	•Application of stringent laws to stop global warming and preserve biodiversity. •Development of new policies to provide sustainable living conditions to all. •Adequate preparations to deal with hindrance caused by extreme weather in patient and doctor transportation, regular dialysis facilities. •Provision for screening, vaccine and treatment facilities for infectious diseases particularly in tropical and sub-tropical regions.
**Political and legal factors**	•Direct and indirect impact on kidney outcomes as influence universal health care and health care system, promote gender and racial equality and prevent malpractice.	•Government policies for dialysis and renal transplantation. •Development of legal framework to provide clean water supply, housing conditions, education and ensure equitable health care •Law enforcement againstorgan trading

AKI, Acute kidney injury; CKD, Chronic kidney diseases; SES, Socioeconomic status; SDoH, Social determinants of health.

## Summary and conclusions

SDoH are the circumstances that have a large impact on an individual’s well-being and health. Variables such as socio-economic status, racial/gender discrimination, community support, and environmental conditions in conjecture with legal and political factors play a key role in influencing a person’s health. They are crucial determinants of the quality of life in CKD patients, especially in low-resource settings. SDoH has the potential to modulate the occurrence, prevalence, progression, and management of kidney disease Deliberations on the complex interplay of these factors are prudent, as addressing these affairs shall enhance the outcomes of kidney disease patients. Public health care providers should unite with the policymakers to develop health-promotion strategies and make them reach the individual level maintaining equity in health care delivery. Finally, further research to facilitate a better understanding of SDoH and their implications on kidney health is warranted to design novel interventions.

## Author contributions

UA was responsible for the conceptualizing the manuscript and for writing up the sections of social determinants of Health (along with PM) PM wrote the social determinants of health and the final corrections were done by her. SK contributed to the political determinants of health section VL was the senior author whose ideas were incorporated in writing this manuscript. She went through the whole manuscript and suggested corrections. All authors contributed to the article and approved the submitted version.

## Conflict of interest

The authors declare that the research was conducted in the absence of any commercial or financial relationships that could be construed as a potential conflict of interest.

## Publisher’s note

All claims expressed in this article are solely those of the authors and do not necessarily represent those of their affiliated organizations, or those of the publisher, the editors and the reviewers. Any product that may be evaluated in this article, or claim that may be made by its manufacturer, is not guaranteed or endorsed by the publisher.
